# DACHPt-Loaded Nanoparticles Self-assembled from Biodegradable Dendritic Copolymer Polyglutamic Acid-*b*-D-α-Tocopheryl Polyethylene Glycol 1000 Succinate for Multidrug Resistant Lung Cancer Therapy

**DOI:** 10.3389/fphar.2018.00119

**Published:** 2018-02-21

**Authors:** Hsiang-I Tsai, Lijuan Jiang, Xiaowei Zeng, Hongbo Chen, Zihuang Li, Wei Cheng, Jinxie Zhang, Jie Pan, Dong Wan, Li Gao, Zhenhua Xie, Laiqiang Huang, Lin Mei, Gan Liu

**Affiliations:** ^1^School of Life Sciences, Tsinghua University, Beijing, China; ^2^School of Pharmaceutical Sciences (Shenzhen), Sun Yat-sen University, Guangzhou, China; ^3^Department of Radiation Oncology, Second Clinical Medicine College of Jinan University, Shenzhen Municipal People's Hospital, Shenzhen, China; ^4^Division of Life and Health Sciences, Graduate School at Shenzhen, Tsinghua University, Shenzhen, China; ^5^School of Environmental and Chemical Engineering, Tianjin Polytechnic University, Tianjin, China; ^6^Department of Urology, Affiliated Hospital of Guilin Medical University, Guilin, China

**Keywords:** multidrug resistance, TPGS, dendritic copolymers, nanoparticles, DACHPt

## Abstract

The clinical applications of platinum-based antitumor agents are still largely limited by severe side effects as well as multidrug resistance (MDR). To solve these problems, we developed an 1,2-diaminocyclohexane-platinum(II) (DACHPt)-loaded nanoparticle (NP-TPGS-Pt) by self-assembly of poly(amidoamine)-polyglutamic acid-*b*-D-α-tocopheryl polyethylene glycol 1000 succinate (PAM-PGlu-*b*-TPGS) and DACHPt. NP-TPGS-Pt showed robust stability and pH-responsive DACHPt release profile *in vitro* similar to the PEG-containing nanoparticle (NP-PEG-Pt). Meanwhile, in contrast with NP-PEG-Pt, NP-TPGS-Pt exhibited efficient nanoparticle-based cellular uptake by the Pt-resistant A549/DDP human lung cancer cells and caused much more cytotoxicity than free Oxaliplatin and NP-PEG-Pt. Finally, this NP-TPGS-Pt was proved to perform outstanding inhibition of Pt-resistant tumor growth, much superior than free Oxaliplatin and NP-PEG-Pt. Thus, this NP-TPGS-Pt provides a novel powerful nanomedicine platform for combatting multidrug resistant cancer.

## Introduction

Up to now, platinum-based antitumor agents have been widely used to treat lung cancer, bladder cancer, gastric cancer, and ovarian cancer, etc. (Graham et al., 2000; Klein and Hambley, 2009; Wheate et al., 2010; Han and Smith, 2013; Harrach and Ciarimboli, 2015; Torre et al., 2015; Ma et al., 2017). However, their clinical outcomes are still largely limited by severe side effects (Nishiyama et al., 2001; Xiao et al., 2017) and multidrug resistance (Wang and Lippard, 2005; Li et al., 2015; Yin et al., 2017). Drug nanocarriers, which can prolong the *in vivo* half-life of drugs and promote the enrichment in solid tumors through the enhanced permeation and retention (EPR) effect, have been verified potentially applicable to antitumor therapy (Peer et al., 2007; Oberoi et al., 2013; Salomone, 2013; Wang and Guo, 2013; Huang et al., 2015; Xu et al., 2015; Li et al., 2017; Liu et al., 2017b). We have previously reported an unimolecular micelle (UM/DACHPt) prepared by loading antitumor agent 1,2-diaminocyclohexane-platinum(II) (DACHPt) with dendritic block copolymer PAM-PGlu-*b*-PEG (Liu et al., 2017a). Compared to micelles self-assembled from block copolymer, this micelle showed superior stability, thereby extending the *in vivo* half-life, enhancing antitumor effects and reducing side effects. Nevertheless, besides relieving side effects, platinum drug-loaded nanocarriers always need to meet the requirement for overcoming tumor multidrug resistance. Since the antitumor effects of unimolecular micelles are inferior to those of free drugs *in vitro*, they would hardly fulfill the requirement. Therefore, it is of great significance to optimize the nanocarrier structure to be both stable *in vivo* and multidrug-resistant.

As a soluble derivative of vitamin E, D-α-tocopheryl polyethylene glycol 1000 succinate (TPGS) is esterified from the acid group of vitamin E succinate and polyethylene glycol (PEG) 1000. It has been approved by FDA as a safe pharmaceutical excipient (Mu and Feng, 2003; Zhang et al., 2012; Guo et al., 2013; Tan et al., 2017). It is well-documented that TPGS could enhance cellular uptake (Zhang and Feng, 2006; Zeng et al., 2013), and inhibit P-glycoprotein to circumvent drug resistance via interfering with the structure and function of mitochondria (Dintaman and Silverman, 1999; Zhu et al., 2014; Wang et al., 2015; Bao et al., 2017). In addition, our previous work has reported that surface modification of PLGA nanoparticles (NPs) with TPGS prolonged the half-life of drugs *in vivo* and facilitated their cellular uptake (Zeng et al., 2013). Thus we ensure that conjugating TPGS with PAM-PGlu rather than PEG would not only maintain the stability of nanocarrier, but also enhance the cellular uptake and overcome drug resistance. As far as we know, no such PAM-PGlu-*b*-TPGS was reported to prepare DACHPt-loaded nanoparticles.

**Figure 1:**
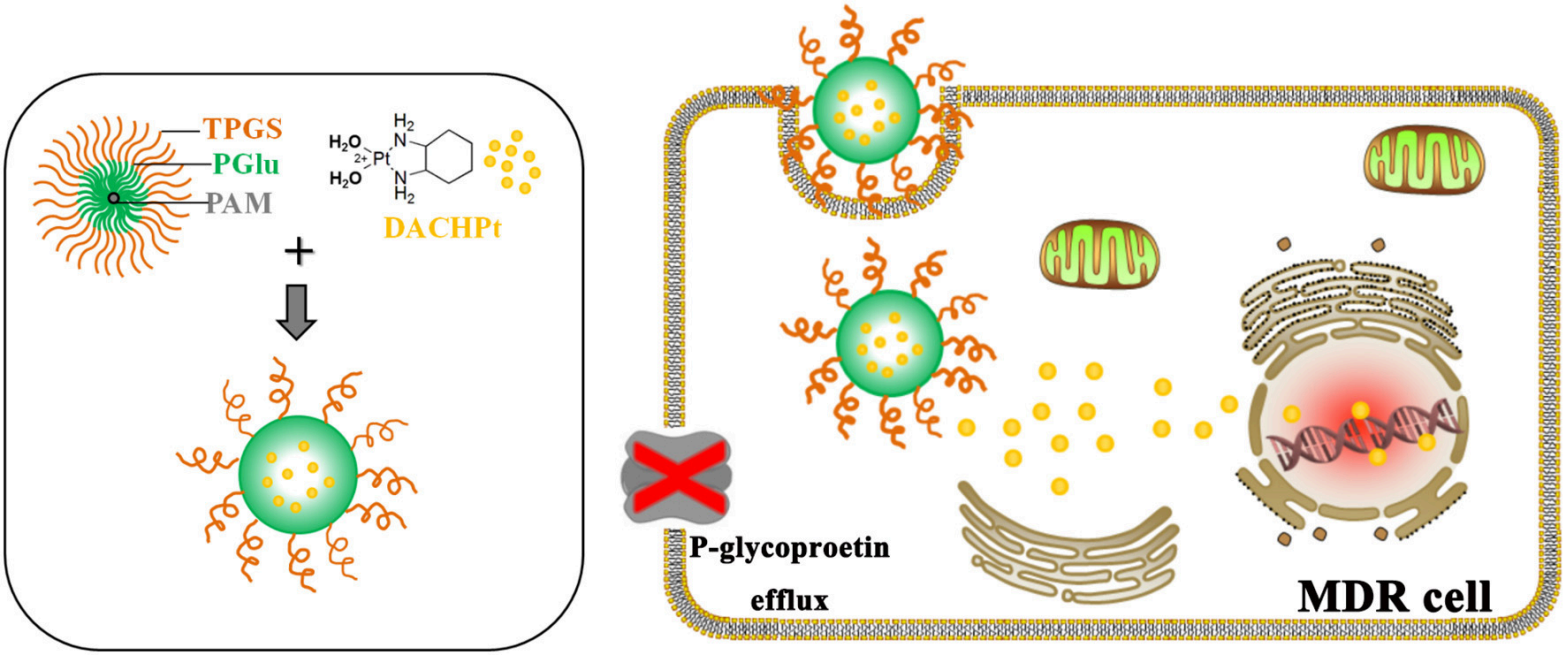
Schematic illustration of overcoming the resistance of A549/DDP cells by NP-TPGS-Pt.

Thus, we designed a novel TPGS-containing dendritic polymer PAM-PGlu-*b*-TPGS to prepare DACHPt-loaded NP-TPGS-Pt. PAM-PGlu-*b*-TPGS consisted of dendritic molecule PAMAM-G3, DACHPt-chelating agent PGlu and polymer TPGS (Figure 1). We then determined the size, zeta potential, drug loading content, encapsulation efficiency, *in vitro* stability and drug release behaviors of NP-TPGS-Pt. Meanwhile, the cellular uptake and *in vitro* cytotoxicity of NP-TPGS-Pt were evaluated by using non-small cell lung cancer cell line A549 and resistant A549/DDP cell line. Finally, the antitumor effects of NP-TPGS-Pt on the A549/DDP model were assessed.

## Experimental section

### Materials

Carboxyl-terminated D-α-tocopheryl polyethylene glycol 1000 succinate (cTPGS) was synthesized in our previous work [21]. BLG-NCA was purchased from Kangmanlin chemicals (Nanjing, China). PAMAM-NH_2_-G3 (PAM-NH_2_, *M*_w_ = 6900 Da), CF_3_COOH, HBr/HAc (w/V 33%), DACHPtCl_2_ and Oxaliplatin were purchased from Aladdin Industrial (Shanghai, China). All the chemicals were commercially available and used as received. A549 and A549/DDP cells were obtained from American Type Culture Collection (ATCC). Fetal bovine serum (FBS), RPMI1640 media and penicillin/streptomycin were both purchased from Gibco. Female Balb/C nude mice (~18 g, 7 weeks old) were purchased from Guangdong Province Medical Animal Center and fed in a SPF (specific pathogen free) class experimental animal room. This study was carried out in accordance with the recommendations of the Care and Use of laboratory animals of Tsinghua University, the Administrative Committee on Animal Research in Tsinghua University. The protocol was approved by the Administrative Committee on Animal Research in Tsinghua University.

### Synthesis of dendritic block copolymer PAM-PGlu-*b*-TPGS

**Figure 2:**
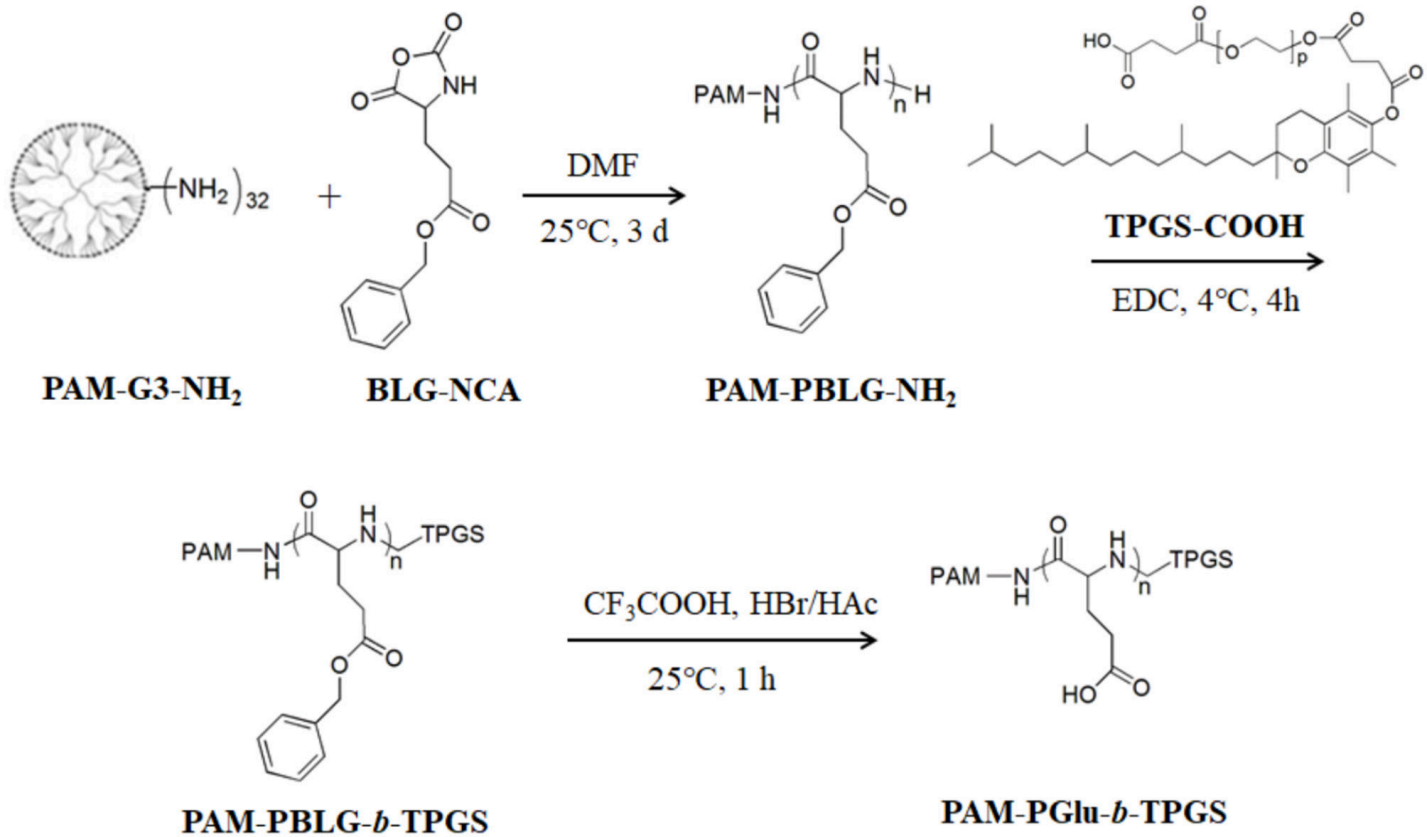
Synthesis of the dendritic block copolymer PAM-PGlu-*b*-TPGS.

As shown in Figure 2, firstly, PAM-PBLG_384_-NH_2_ was synthesized according to our previous work (Liu et al., 2017a). Then PAM-PBLG-NH_2_ (1 g) was reacted with excess TPGS-COOH and EDC at 4°C for 2 h to obtain the dendritic block copolymer PAM-PBLG-*b*-TPGS. The degree of polymerization of TPGS in PAM-PBLG-NH_2_ was verified by ^1^H-NMR spectroscopy (Varian UNITY-plus 400 M nuclear magnetic resonance spectrometer, solvent: CDCl_3_). After that, 20 mL trifluoroacetic acid dissolving PAM-PBLG-*b*-TPGS was added in 2 mL hydrogen bromide (HBr) (33% in acetic acid) and stirred for 1 h at room temperature. Then the solution was neutralized by sodium hydroxide (NaOH) and dialyzed against distilled water (DI water) using a dialysis membrane with molecular weight cutoff (MWCO) of 5 kDa. The aqueous solution of purified product was lyophilized to obtain PAM-PGlu-*b*-TPGS.

### Preparation of DACHPt-loaded nanoparticles

DACHPt-loaded nanoparticles were prepared similar to our previous work. Briefly, PAM-PGlu-*b*-TPGS solution was separately mixed with the DACHPt aqueous solution (Molar ratio Glu/ DACHPt = 3/1) and reacted for 72 h. The formed nanoparticles were purified by ultrafiltration using Centricon Plus-20 centrifugal filter units (MWCO 50 KDa, Millipore, MA, USA).

### Characterization of DACHPt-loaded nanoparticles

The size distribution and zeta potential of DACHPt-loaded nanoparticles were measured using Zetasizer Nano ZS90 (Malvern Instruments Ltd., UK). Before measurement, the freshly prepared nanoparticles were diluted as needed. All measurements were carried out at 25°C. The data were obtained as the average of three measurements.

The morphology of nanoparticles were then observed by transmission electron microscopy (TEM, Tecnai G2 20, FEI Company, Oregon, USA). One microliter of the sample solution was placed on the resulting grids and dried in the air.

To measure the drug loading content (LC) and drug encapsulation efficiency (EE) of the DACHPt-loaded nanoparticles, a predefined amount of nanoparticles were dissolved in 1 mL water. The Pt concentration in the nanoparticles was quantified by Inductively coupled plasma mass spectrometry (ICP-MS, Xseries II, Thermoscientific, USA). The LC and EE of DACHPt-loaded nanoparticles were calculated according to

$$\text{LC}\left(\%\right)=\frac{\text{amout}\;\text{of}\;\text{DACHPt}\;\text{in}\;\text{the}\;\text{nanoparticles}}{\text{amount}\;\text{of}\;\text{the}\;\text{nanoparticles}}\times 100\%$$

$$\text{EE}\left(\%\right)=\frac{\text{amount}\;\text{of}\;\text{DACHPt}\;\text{in}\;\text{the}\;\text{nanoparticles}}{\text{amount}\;\text{of}\;\text{feeding}\;\text{DACHPt}}\times 100\%$$

### *In vitro* drug release study

The stability of NP-TPGS-Pt in cell culture media including 10% FBS at 25°C was evaluated by DLS. The DACHPt release profile of the nanoparticles was monitored by the dialysis method. Firstly, 1 ml of NP-TPGS-Pt was placed into the dialysis bag (MWCO 5 kDa) and immersed in 30 ml of cell culture media including 10% FBS (pH 7.4 and 5.5) at 37°C. The media outside was taken out at defined periods, and the concentrations of Pt were measured by ICP-MS.

### Cellular uptake study

In this study, 5-Aminofluorescein-labeled NP-PEG-Pt, and NP-TPGS-Pt as the model fluorescent probe to investigate the uptake by A549 cells and A549/DDP cells. A549 cells and A549/DDP cells were cultured as described previously (Liu et al., 2017a). The cellular uptake experiments of 5-Aminofluorescein-NPs were performed using CLSM. A549 cells and A549/DDP cells (1^*^10^5^ cells/well) were seeded in 12-well culture plates and cultured overnight in RPMI1640 with 10% FBS. On the following day, the cells were washed once with PBS and incubated with 5-AF-NP-PEG-Pt, and 5-AF-NP-TPGS-Pt in 10% serum containing the media for 3 h at 37°C. The cells were observed using a CLSM (Olympus Fluoview FV-1000, Tokyo, Japan) using an imaging software. The images of the cells were captured with differential interference contrast channel, and the images of 5-Aminofluorescein-NPs and the nuclei of DAPI-stained cells were recorded with following channels: blue channel (DAPI) excited at 340 nm and green channel (5-Aminofluorescein) excited at 488 nm.

### MTT assay

The 50% growth inhibitory concentrations (IC_50_) of free Oxaliplatin, NP-PEG-Pt, and NP-TPGS-Pt in the A549 and A549/DDP cells were measured by the MTT assay. After incubated in a 96-well culture plate (10^4^ cells/well) for 24 h, A549 cells were then exposed to 10, 20, 40, 100, and 200 μM oxaliplatin or DACHPt-loaded nanoparticles (on a platinum basis) for another 24, 48, and 72 h. At each point of time, The MTT solution was added, and cell viability was measured in a Bio-Rad 680 microplate reader by formazan absorbance at 490 nm.

### *In vivo* antitumor efficacy

After randomly divided into four groups (*n* = 5), the nude mice were built with human A549 xenograft tumor model by injection of 1.5 × 10^6^ A549/DDP cells (100 μL) subcutaneous at the right axilla of each mouse. Before initiating treatment, tumors were observed frequently and allowed to grow to ~50 mm^3^ in volume. Mice were injected intravenously five times via tail vein on days 0, 4, 8, 12, and 16 with saline, Oxaliplatin and DACHPt-loaded nanoparticles (6 mg/kg on a Pt basis). The antitumor efficacy was determined in accordance with the tumor volume (V), which was calculated similar to our previous work. The body weights of mice were simultaneously measured to evaluate the systemic toxicity. The results were considered statistically significant if two-tailed *P*-values were < 0.05.

### Statistical analysis

All of the experiments were carried out at least three times. The Data are expressed as mean ± *SD* unless noted otherwise and analyzed for significance using Student's *t*-test. Probability value (*P*) < 0.05 indicates statistically significant. ^*^P < 0.05; ^**^P < 0.01.

## Results and discussion

### Synthesis and characterizations of dendritic copolymer PAM-PGlu-*b*-TPGs

**Figure 3:**
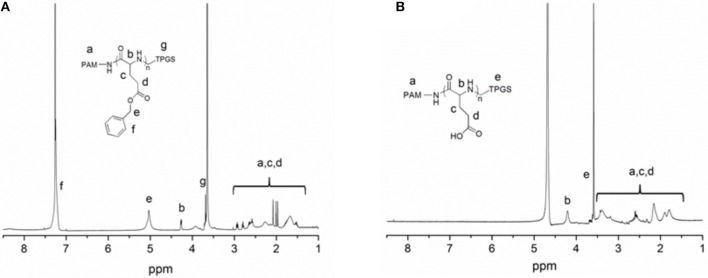
**(A)** ^1^H-NMR spectra of PAM-PBLG_384_-NH_2_ in CDCl_3_, and **(B)** PAM-PGlu_384_-*b*-(TPGS)_30_ in D_2_O.

The chemical structure of dendritic copolymer PAM-PGlu-*b*-TPGS was validated by ^1^H NMR spectroscopy. Figure 3A shows the ^1^H NMR spectrum of PAM-PBLG-*b*-TPGS, in which a, b, c, d, e, and g represent the characteristic peaks of methylene group (CH_2_) in PAM, -C(O)C*H*(CH_2_)NH-, -CHC*H*_2_CH_2_C(O)-, -CHCH_2_C*H*_2_C(O)- and C_6_H_5_C*H*_2_- in PBLG, as well as CH_2_ of PEG in TPGS, demonstrating the successful linking of TPGS. The number of TPGS in each dendritic molecule was 30 by calculating the areas of peaks e and g. As exhibited in Figure 3B, the characteristic peak of benzyl group in BLG disappears, suggesting that the deprotection was successful and PAM-PGlu-*b*-TPGS had been successfully prepared.

**Figure 4:**
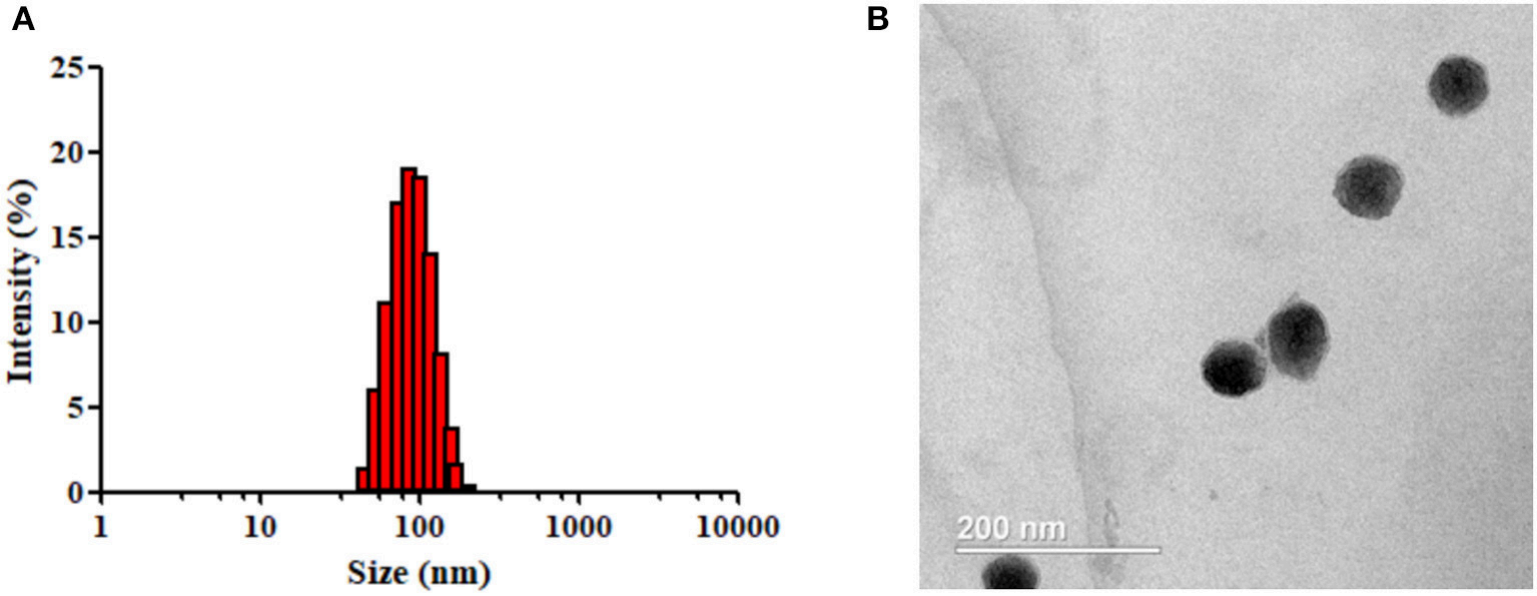
**(A)** Size distribution and **(B)** TEM image of NP-TPGS-Pt.

Afterwards, we obtained NP-TPGS-Pt by complexing PAM-PGlu-*b*-TPGS with DACHPt. The NPs had a narrow monodisperse distribution and the average hydrodynamic diameter of ~85.3 nm (Figure 4A). TEM presented that NF-TPGS-Pt were uniformly distributed spherical particles with the size of about 60 nm, being consistent with the DLS results (Figure 4B). In the meantime, we prepared NP-PEG-Pt with PAM-PGlu-*b*-PEG and DACHPt as control (Table 1).

**Table 1:** Characterization of DACHPt-loaded nanoparticles.

**NPs**	**Dh (nm)**	**PDI**	**ZP (mV)**	**LC (%)**	**EE (%)**
NP-TPGS-Pt	85.3 ± 3.6	0.16	−16.3 ± 1.7	26.3 ± 1.5	75 ± 3.7
NP-PEG-Pt	55.3 ± 2.4	0.13	−20.3 ± 2.0	24.3 ± 1.1	71 ± 3.4

*PDI, polydispersity index; ZP, zeta potential; LC, loading content; EE, encapsulation efficiency*.

### Stability and drug release study

**Figure 5:**
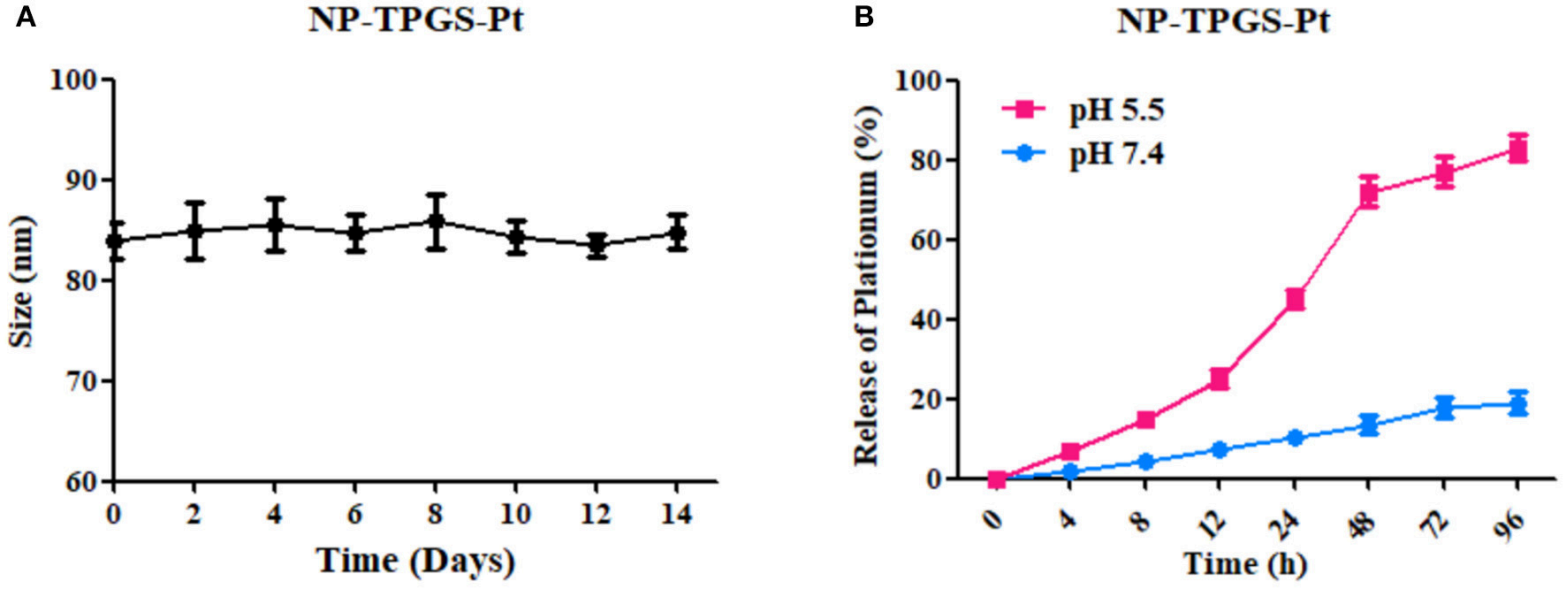
**(A)** DACHPt-loaded NPs incubated in medium containing 10% FBS maintained their sizes for 14 days. **(B)** Accumulative release of DACHPt-loaded NPs in media containing 10% FBS with different pH-values. The result was reported as the average of three measurements.

Then we determined the *in vitro* stability of NP-TPGS-Pt. The size of NP-TPGS-Pt remained stable after 14 consecutive days of culture in cell medium (Figure 5A), indicating that it holds robust stability.

As evidenced by ICP-MS, the drug loading content of NP-TPGS-Pt was 26.3% (Table 1). Subsequently, we tested the *in vitro* release of DACHPt from NP-TPGS-Pt. In a previous study, we found that Pt release was accelerated in a chloride ion-containing environment under acidic conditions. Thus, we herein compared such release from NP-TPGS-Pt at pH 7.4 and 5.5. At pH 7.4, the drug release rate from NP-TPGS-Pt was only 20% after 96 h (Figure 5B), revealing that these NPs were fairly stable under physiological conditions and drug hardly leaked. On the other hand, NP-TPGS-Pt managed to rapidly release DACHPt at pH 5.5, predicting that they may work so inside tumor cells.

### Cellular uptake

**Figure 6:**
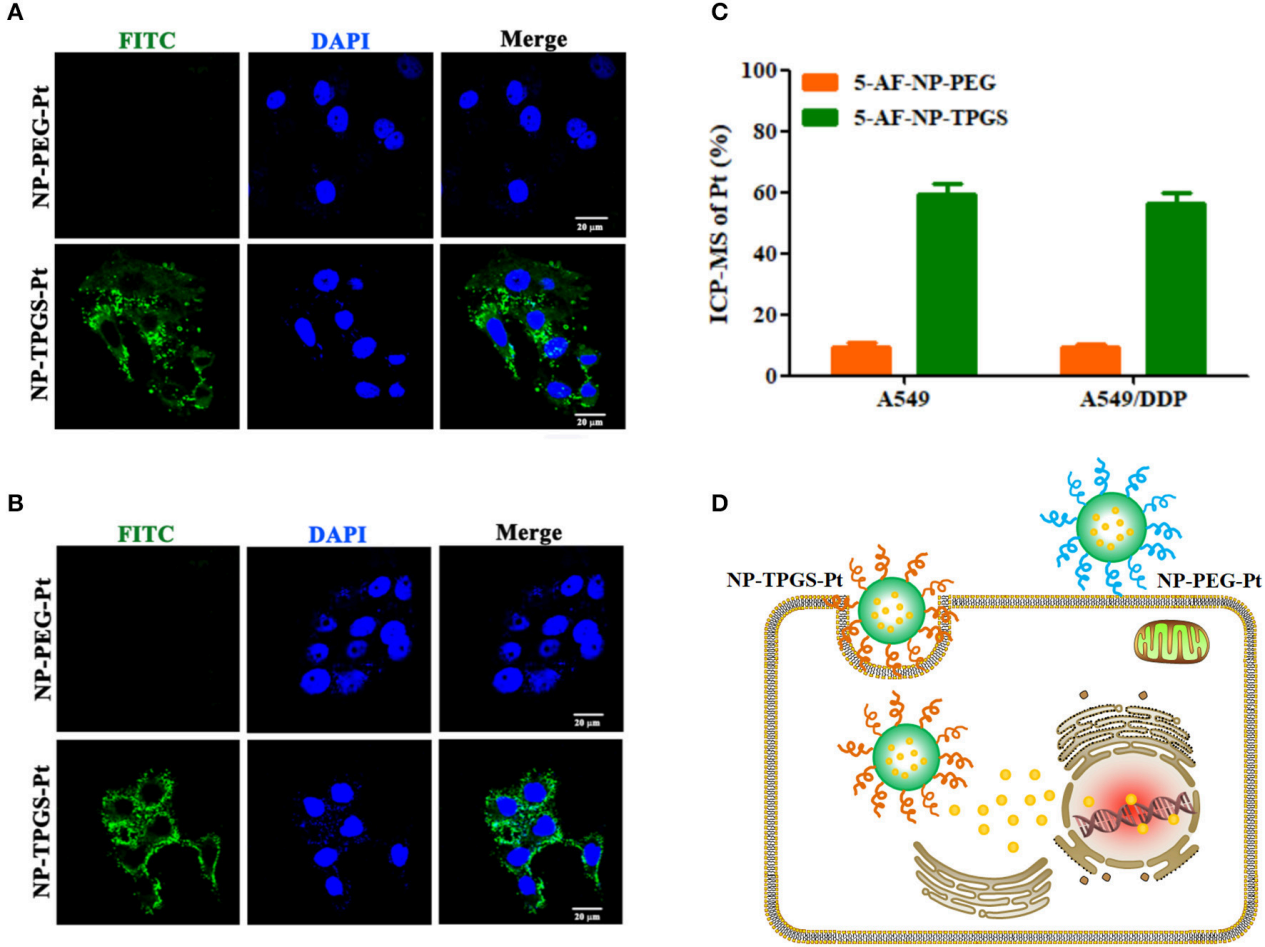
Cellular uptake of NP-TPGS-Pt by A549 andA549/DDP cells. **(A,B)** CLSM images in A549 and A549/DDP cells, **(C)** Cellular uptake efficiency of A549 and A549/DDP cells after incubation with NP-PEG-Pt and NP-TPGS-Pt for 3 h. **(D)** An illustration for cell uptake in A549/DDP cells. (Scale bar: 20 μm).

It is well-established that drug delivery into cells through uptake of nanocarrier can well escape efflux protein-mediated transport, thereby overcoming multidrug resistance (Wei et al., 2015). Accordingly, we first studied the cellular uptake of NP-TPGS-Pt with confocal laser scanning microscopy (CLSM). We covalently linked NP-PEG-Pt and NP-TPGS-Pt with 5-aminofluorescein (AF), and observed their endocytosis by A549 and A549/DDP cells. After 3 h, A549 cells emitted obvious fluorescence of NP-TPGS-Pt-AF (green), but the NP-PEG-Pt-AF group barely showed signal (Figure 6A), because intracellular fluorescence originated from NPs to which AF was covalently linked. Collectively, TPGS modification substantially promoted the cellular uptake of nanocarrier, whereas PEG hardly underwent uptake due to the apparent shielding effect. Given that the fluorescence signal of AF (green) in A549/DDP cells was almost equivalent to that of A549 cells, NP-TPGS-Pt was also subjected to uptake by A549/DDP cells (Figure 6B).

Then we further quantified the cellular uptake efficiency of NP-TPGS-Pt at 3 h. As presented in Figure 6C, the NP-PEG-Pt uptake efficiencies of A549 and A549/DDP cells are lower than 20%, while those for NP-TPGS-Pt reach as high as 60%. In short, drug-resistant cells were still capable of efficient uptake of NP-TPGS-Pt (Figure 6D).

### *In vitro* cytotoxicity

**Figure 7:**
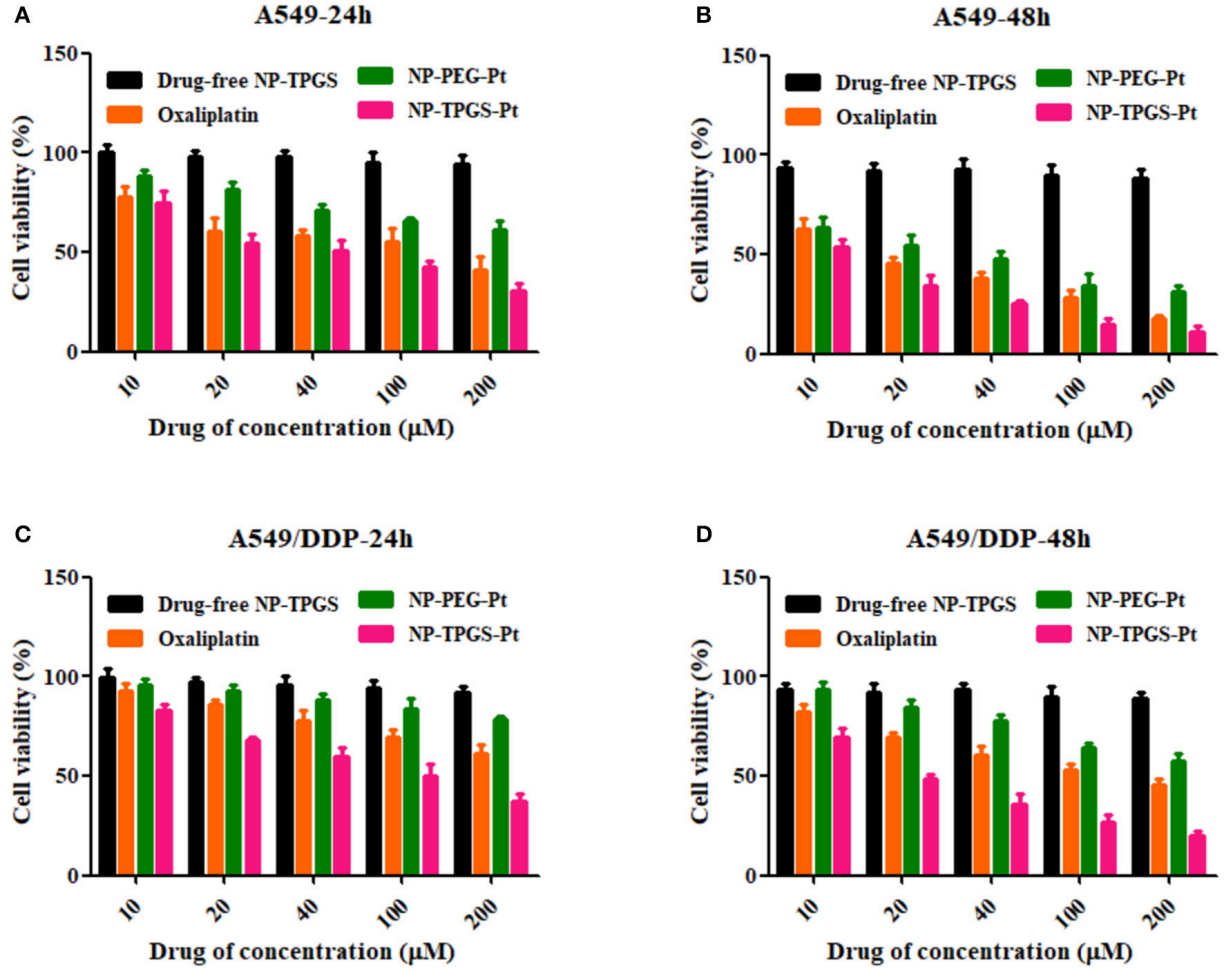
Cell viability of A549 and A549/DDP cells incubated with the TPGS/DACHPt compared with that of oxaliplatin, NP-PEG-Pt and NP-TPGS-Pt at the same Oxaliplatin dose and that of the drug-free TPGS with the same polymer concentrations: **(A,B)** A549 cells for 24 and 48 h. **(C,D)** A549/DDP cells for 24 and 48 h.

To demonstrate the ability of NP-TPGS-Pt to kill tumor cells, especially the drug-resistant ones, we performed MTT assay using A549 and A549/DDP cells. Meanwhile, free Oxaliplatin, NP-PEG-Pt and drug-free NP-TPGS were employed as controls. A549 and A549/DDP cells were treated by different concentrations of drugs for 24 and 48 h. After 24 h of culture, Oxaliplatin significantly killed A549 cells, whereas NP-PEG-Pt also exerted much weaker effects (Figure 7A), being in agreement with the above cellular uptake efficiencies. However, NP-TPGS-Pt worked more effectively than Oxaliplatin did. Moreover, the IC_50_ values of Oxaliplatin and NP-PEG-Pt were 98.8 and 214.0 μM, respectively, but that of NP-TPGS-Pt was merely 45.4 μM (Table 2). With increasing culture time, although the antitumor effects of the three Oxaliplatin formulations were significantly boosted, the outcomes of NP-TPGS-Pt were significantly superior to those of Oxaliplatin and NP-PEG-Pt (Figure 7B). Taken together, modifying NP surface with TPGS increased the cytotoxicity through efficient uptake and persistent drug release.

**Table 2:** IC_50_ values of Oxaliplatin, NP-PEG-Pt and NP-TPGS-Pt against A549 and A549/DDP cells following 24 and 48 h of incubation.

	**Time (h)**	**IC_50_ (μM)**		
		**Oxaliplatin**	**NP-PEG-Pt**	**NP-TPGS-Pt**
A549	24	98.8 ± 8.4	214.0 ± 2.0	45.4 ± 2.7
	48	16.1 ± 1.1	31.1 ± 2.6	9.8 ± 0.8
A549/DDP	24	347.4 ± 10.2	–	85.4 ± 8.7
	48	118.6 ± 9.8	235.2 ± 10.8	23.1 ± 3.5

On the other hand, Oxaliplatin killed A549/DDP cells far less effectively than A549 cells. Since the IC_50_ values of Oxaliplatin at 24 and 48 h were 347.4 and 118.6 μM, respectively, A549/DDP cells were indeed strongly drug-resistant. Likewise, the antitumor effects of NP-PEG-Pt were inferior to those of Oxaliplatin. In contrast, the IC_50_ values of NP-TPGS-Pt at 24 and 48 h were 85.4 and 23.1 μM, respectively which were much lower than those of Oxaliplatin (Figures 7C,D), and slightly lower than those against A549 cells. Hence, NP-TPGS-Pt could overcome the drug resistance of cells. Additionally, MTT assay disclosed no influence of DACHPt-free NP-TPGS on cell growth, so they were highly biosafe and thus applicable to practice.

### *In vivo* antitumor effects

**Figure 8:**
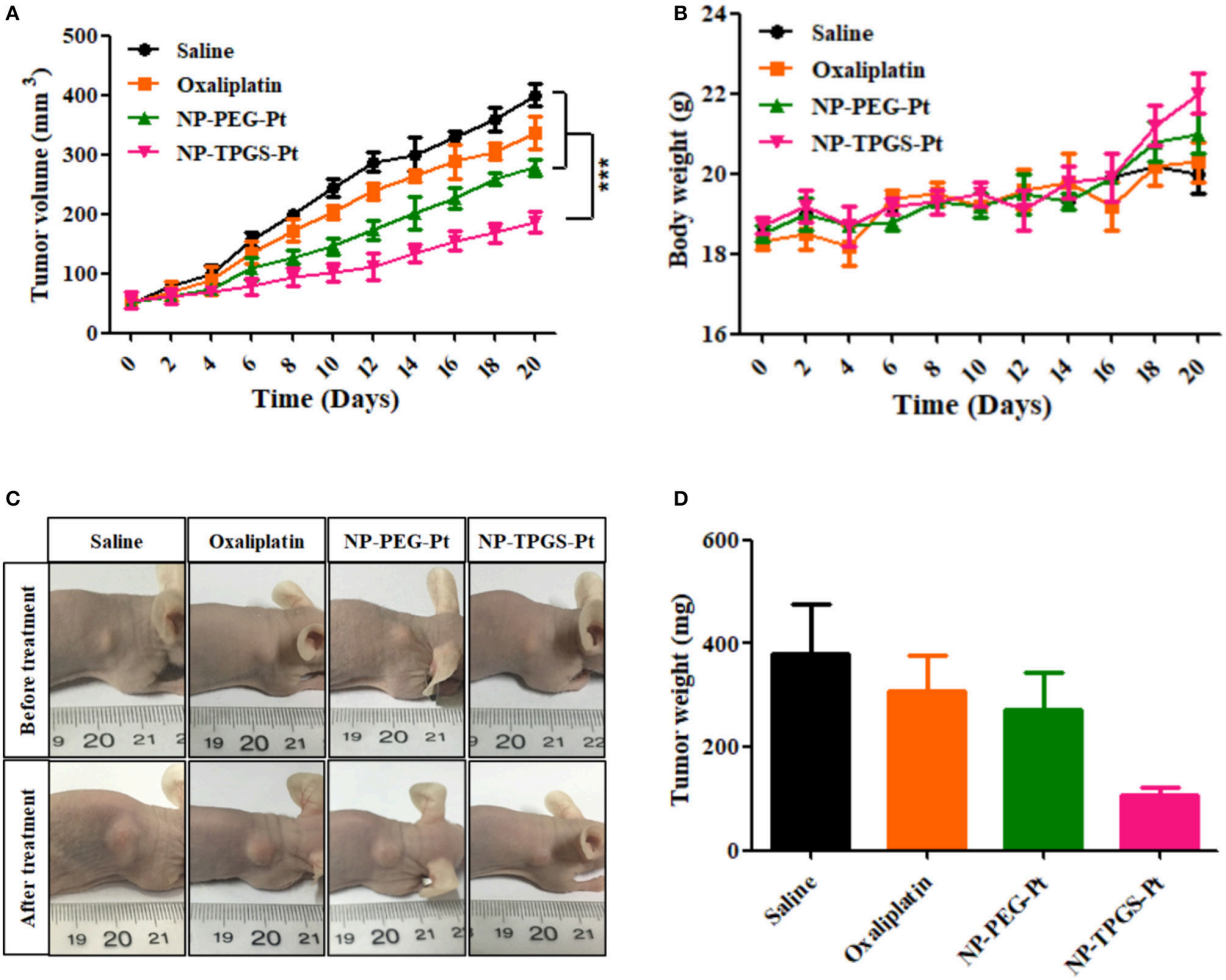
Influence of DACHPt formulations on antitumor effects **(A)**, and changes of body weight **(B)** of A549/DDP xenograft-bearing nude mice. Images **(C)** and weights **(D)** of tumors resected from each group of sacrificed mice on the last day.

Given the positive results above, we ultimately evaluated the *in vivo* antitumor effects of NP-TPGS-Pt. The mice xenografted with A549/DDP cells were injected with normal saline, Oxaliplatin, NP-PEG-Pt and NP-TPGS-Pt respectively every 4 days, five times in total. After 20 days of treatment, the average tumor volumes of saline, Oxaliplatin and NP-PEG-Pt groups grew to 408.8, 337.3, and 278.8 mm^3^, respectively, but that of the NP-TPGS-Pt group only increased to 188.7 mm^3^ (Figure 8A). Clearly, NP-TPGS-Pt markedly inhibited tumor growth, with better outcomes than those of Oxaliplatin and NP-PEG-Pt. Besides, the body weights of NP-TPGS-Pt-treated mice and the other three groups were similar, without obvious changes (Figure 8B). After 20 days of treatment, all mice were sacrificed, from which tumors were collected. Figures 8C,D show the morphologies and average body weights of all tumors, both confirming the outstanding antitumor effects of NP-TPGS-Pt. Overall, NP-TPGS-Pt were able to solve tumor multidrug resistance.

## Discussion and conclusions

In our previous research (Liu et al., 2017a), we developed the DACHPt-loaded UM (UM/DACHPt) dendritic block copolymer for micelles formulation of small molecular anti-tumor drugs and characterized the properties of the nanoparticle *in vitro*. Since the antitumor effects of unimolecular micelles are inferior to those of free drugs *in vitro*, they would hardly fulfill the requirement. Therefore, to optimize the nanocarrier structure to be both stable *in vivo* and multidrug-resistant. Indeed, TPGS has been reported to inhibit P-glycoprotein mediated multi-drug resistance (MDR) in tumor cells, which may reduce the excretion of drugs (Collnot et al., 2007; Zeng et al., 2013).

From the DLS results (Figure 4A and Table 1), we obtained NP-TPGS-Pt by complexing PAM-PGlu-b-TPGS with DACHPt. The NPs had a narrow monodisperse distribution and the average hydrodynamic diameter of ~85.3 nm. The size of NP-TPGS-Pt was much larger than which of NP-PEG-Pt. Because of PEG is completely hydrophilic polymers, PEG-b-PGlu could interact with DACHPt to be unimolecular micelles, therefore the size and the dendritic block copolymers itself size didn't big changed. But TPGS has certain hydrophobic, after PEG-b-PGlu interact with DACHPt, TPGS would induce the accumulation of the dendritic block copolymers, so the final particle size of NP-TPGS-Pt was bigger than NP-PEG-Pt. In other reports also showed TPGS dendritic block copolymers gathered into 100 nanometers in micelle (Zeng et al., 2013). And in TEM presented that NF-TPGS-Pt were uniformly distributed spherical particles with the size of about 60 nm, being consistent with the DLS results (Figure 4B). The particle size measured by TEM was smaller than that by DLS, because DLS detected the hydrodynamic diameter in aqueous solution in which the hydrophilic TPGS layer expanded while TEM was performed by using dry NPs.

Previous reports found that Pt release was accelerated in a chloride ion-containing environment under acidic conditions (Song et al., 2012). In Figure 5, the drug release rate from NP-TPGS-Pt was only 20% after 96 h, revealing that these NPs were fairly stable under physiological conditions and drug hardly leaked. We observed the acid environment could accelerate the Pt release, because the accelerated release at acidic pH may be due to the protonation of carboxylic groups of PGlu, which weakens the drug and micelles coupling.

In conclusion, we successfully synthesized the dendritic block copolymer PAM-PGlu-*b*-TPGS which was thereafter self-assembled into nanoparticles by chelating the potent antitumor agent DACHPt. This TPGS-coated nanocarrier had robust stability and underwent nanoparticle-based cellular uptake by drug-resistant cancer cells, eventually remarkably suppressing the growth of these cells and drug-resistant tumors *in vivo*. Hence, the nanocarrier provides a novel strategy for treating multidrug-resistant tumors.

## Author contributions

GL, LH, and ZL designed the research project; H-IT and LJ had full controlled the experiments, data analysis, and preparation of article; XZ, HC, WC, JZ, JP, DW, LG, ZX, and LM were involved in planning the analysis and drafting the article. The final draft article was approved by all the authors.

### Conflict of interest statement

The authors declare that the research was conducted in the absence of any commercial or financial relationships that could be construed as a potential conflict of interest.
